# *Musa paradisiaca* L. leaf and fruit peel hydroethanolic extracts improved the lipid profile, glycemic index and oxidative stress in nicotinamide/streptozotocin‐induced diabetic rats

**DOI:** 10.1002/vms3.389

**Published:** 2020-12-05

**Authors:** Osama M. Ahmed, Sanaa M. Abd El‐Twab, Hessah M. Al‐Muzafar, Kamal Adel Amin, Sarah M. Abdel Aziz, Mohammed Abdel‐Gabbar

**Affiliations:** ^1^ Physiology Division Zoology Department Faculty of Science Beni‐Suef University Beni‐Suef Egypt; ^2^ Experimental Obesity and Diabetes Research Lab Faculty of Science Beni‐Suef University Beni‐Suef Egypt; ^3^ Department of Chemistry College of Science Imam Abdulrahman Bin Faisal University Dammam Saudi Arabia; ^4^ Biochemistry Department Faculty of Science Beni‐Suef University Beni‐Suef Egypt

**Keywords:** heart function, kidney function, lipid profile, *Musa paradisiaca*, NA/STZ‐induced diabetes

## Abstract

This study aimed to assess antihyperlipidemic, cardiac and antioxidant effects as well as mode of actions of *Musa paradisiaca* (*M. paradisiaca*) leaf and fruit peel hydroethanolic extracts in nicotinamide (NA)/streptozotocin (STZ)‐induced diabetic rats. Experimental diabetes mellitus was induced by a single intraperitoneal injection of STZ (60 mg/kg body weight), 15 min after intraperitoneal injection of NA (120 mg/kg body weight). NA/STZ‐induced diabetic rats were orally supplemented with *M. paradisiaca* leaf and fruit peel hydroethanolic extracts in a dose of 100 mg/kg body weight/day for 28 days. The treatment of NA/STZ‐induced diabetic rats with *M. paradisiaca* leaf and fruit peel extracts significantly decreased the elevated fasting and post‐prandial serum glucose, total cholesterol, triglycerides, LDL‐cholesterol and vLDL‐cholesterol levels and significantly increased the lowered serum insulin level, liver glycogen content, serum HDL‐cholesterol level, homeostasis model assessment‐insulin resistance (HOMA‐IS) and HOMA‐β cell function. The elevated cardiovascular risk indices in diabetic rats were significantly improved due to treatment with *M. paradisiaca* extracts. Concomitant with the increase in liver glycogen content, the glucose‐6‐phosphatase activity significantly decreased reflecting the decrease in hepatic glucose output. The heart function was potentially ameliorated as manifested by decrease in the elevated serum creatine kinase‐MB, lactate dehydrogenase and aspartate aminotransferase activities after treatments of diabetic rats with *M. paradisiaca* extracts. The elevated liver lipid peroxidation and the decline in liver glutathione content and superoxide dismutase, glutathione peroxidase and glutathione‐S‐transferase activities were significantly reversed by treatments. Thus, it can be concluded that *M. paradisiaca* leaf and fruit peel hydroethanolic extracts may have antihyperlipidemic and cardioprotective potentials in NA/STZ‐induced diabetic rats. These effects may be mediated *via* improvements in the glycemic state, β‐cell function, tissue insulin sensitivity, and antioxidant defense mechanism.

## 1 INTRODUCTION

Diabetes mellitus (DM) is a syndrome characterized by decreased insulin secretion and/or tissue insulin sensitivity and altered metabolism of carbohydrates, lipids and proteins, in addition to increased cardiovascular risks (Davis et al., 1996). Type 1 diabetes mellitus (T1DM) is characterized by irreversible and autoimmune pancreatic *β*‐cell destruction (Ozen et al., 2020). Type 2 DM (T2DM) is the most incident form, responsible for 90% of the disease prevalence (Wild et al., 2004; World Health Organization, 2006). The progression of T2DM includes the development of impaired glucose tolerance, reduced tissue insulin sensitivity and eventual dysfunction of β‐cells in the pancreatic islets (Cersosimo et al., 2000; Parikh et al., 2007). Nicotinamide (NA)/streptozotocin (STZ)‐induced DM is most commonly used as animal model of T2DM in rats to validate the effect and to scrutinize the mechanisms of action of new therapeutic agents. In this model, there are both moderate impairment in insulin secretion and insulin resistance, which is a characteristic feature of T2DM (Ali et al., 2020; Gorinstein et al., 2007; Singh & Singh, 2010; Zhou et al., 2009).

T2DM and its associated hyperglycemia and hyperlipidemia lead to numerous acute and chronic complications. They increase risks for nephropathy and cardiovascular diseases (Braunwald, 2019). Many studies have indicated that DM is associated with excess production of reactive oxygen species (ROS) (Peerapatdit et al., 2017, 2019, 2017, 2006; Ahmed, et al., 2017; Ahmed, et al., 2019; Ahmed, et al., 2017; Saravanan & Ponmurugan, 2011), which in turn, is implicated in the pathogenesis of many diabetic complications including atherosclerosis, kidney injury and heart failure (Wilcox & Gutterman, 2005). The production of ROS is reduced by an antioxidant defense system which included both non‐enzymatic antioxidants including vitamin C, vitamin D and glutathione (GSH) and ROS‐scavenging antioxidant enzymes such as catalase (CAT), GSH peroxidase (GPx), GSH‐S‐transferase (GST) and superoxide dismutase (SOD) (Adewole et al., 2008; Budin et al., 2009; Tan et al., 2018). Thus, the use of antioxidative agents to enhance the antioxidant defense system in T2DM may be an important issue to prevent or reduce the progress of diabetic complications including nephropathy and cardiomyopathy. In recent years, great interest has been focused on using natural antioxidants due to the possible adverse effects of synthetic antioxidants. Plants and their crude extracts are rich sources of bioactive natural antioxidants.

*Musa paradisiaca* (*M. paradisiaca*) and other related *Musa species* are commonly named as banana in English and Kela in Indian languages and it is a member of the family Musaceae (Ploetz et al., 2007). Traditionally, the plant was revealed to have anti‐diarrheal, anti‐dysentery, anti‐helmentic, anti‐ulcerative, anti‐microbial, anti‐hyperglycemic, anti‐hypertensive, diuretic, anti‐urolithiatic, wound healing, anti‐malarial and anti‐snake venom activities (Abdel Aziz et al., 2020; Imam & Akter, 2011; Laeliocattleya et al., 2018) as well as antioxidant properties (Sidhu & Zafar, 2018; Singh & Prakash, 2015). As *M. paradisiaca* fruit pulps are edible parts but its leaves and peels are usually thrown as waste by‐products in many countries, most past publications on this plant were directed to assess the nutritional significance of fruit pulps. However, in last recent few years, many researchers have interested to assess the pharmacological effects of *M. paradisiaca* leaves and fruit peels because they are also good sources of antioxidant polyphenols and other bioactive compounds (Abdel Aziz et al., 2020; Sathya et al., 2014; Sidhu & Zafar, 2018). Phytochemical analysis in the publication of Kappel et al. (2013) demonstrated the presence of antioxidant flavonoid, rutin, in crude extract and fractions of *M. paradisiaca* leaves as the major compound. It was also reported that alcoholic extract of *M. paradisiaca* peels contains many antioxidant phenolic and flavonoid compounds including ellagic acid, gallic acid, rutin, myricetin and naringenin (Behiry et al., 2019). Furthermore, GC‐MS analysis of *M. paradisiaca* indicated the presence of phytol, octadecatrienoic acid, hexadecanoic acid and octadecadienoic acid as major components in the leaf extract and vitamin E, octadecenamide, *β*‐sitosterol and stigmasterol as major phytochemicals in the fruit peel extract; all of these constituting compounds were reported to have antioxidant activities (Abdel Aziz et al., 2020). In the same regard, the GC‐MS analysis performed by Waghmare et al. (2014) indicated the presence of many antioxidant components including beta‐tocopherol, vitamin E, estragole, hexadecanoic acid ethyl, epicatechin, gallocatechin, p‐coumaric acid ethyl ester and 1,2 benzenedicarboxylic acid mono (2‐ethylhexyl) ester in banana peel ethanolic extract.

In conductance with the previous publications, the current study was undertaken to assess the effects of *M. paradisiaca* leaf and peel hydroethanolic extracts on serum lipid profile, glycemic index, heart function parameters together with oxidative stress and antioxidant defense system in NA/STZ‐induced diabetic Wistar rats.

## 2 MATERIALS AND METHODS

### 2.1 Plant material

*M. paradisiaca* (banana) fruits were obtained from a local market and the leaves were collected from the Banana Agriculture Gardens, Beni‐Suef, Egypt, in the month of May. in the month of May. The plant was authenticated by Dr. Mohamed A. Fadl, Botany Department, Faculty of Science, Beni‐Suef University, Egypt. Plant sample was deposited in the Herbarium of Botany Department of Faculty of Science, Beni‐Suef University, Egypt (Voucher number: BNSU‐1371). By using the NCBI taxonomy database, the taxonomy ID was 89,151 (NCBI: txid89151) (https://www.ncbi.nlm.nih.gov/Taxonomy/).

### 2.2 Preparation of plant extract

After the botanical authentication, the plant leaf was washed under running tap water to remove adhering dust, while fruits were manually peeled, and good‐quality peels were selected. The plant materials were air dried under shade for 5 days and powdered by electric grinder. The resulting powder material was subjected to extraction with 70% ethanol. About 500 g of powdered material of *M. paradisiaca* fruit peels and leaves were separately taken in two large and clean beakers and soaked in adequate volume of ethanol (70%). The beakers were sealed and kept for a period of three day accompanying occasional shaking and stirring at room temperature. The solutions were then filtered through muslin cloth. The filtrate obtained was evaporated to semisolid mass by using rotary evaporator to obtain the hydroethanolic extract. The yield of the hydroethanolic extracts of leaves and fruit peels was, respectively, 2.8% and 3% of dry weight. The viscous green and brown crude extract of leaves and peels were obtained and stored at −20°C till using (Williamson et al., 1996).

### 2.3 Chemicals and equipment

NA (nicotinamide) and STZ [2‐deoxy‐2‐(3‐methyl‐3nitrosoureido)‐D‐glycopyranoside], as a diabetogenic agent, were purchased from Sigma‐Aldrich Chemical Co., St Louis, MO, USA. All other chemicals were of analytical grade and were obtained from standard commercial supplies. Glucose, creatine kinase‐MB (CK‐MB), aspartate aminotransferase (AST), and lipid profile kits were purchased from Spinreact (Spain). Lactate dehydrogenase (LDH) kit was obtained from Stanbio Laboratories (Texas, USA). Insulin Sandwich ELISA kit was purchased from Linco Research (USA). Teflon homogenizer (Glas‐Col, Terre Haute, USA) was used for homogenization of liver. UV spectrophotometer (ERBA Chem 7; ERBA Diagnostics Mannheim GmbH, Mallaustrasse 69–73 68,219 Mannheim, Germany) was used for detection of biochemical parameters and oxidative stress parameters. Cooling centrifuge (Centurion Scientific K3 Series Centrifuges; Core Life Sciences, Irvine, 92,623 California, USA) was used for centrifugation of blood and liver homogenate. ELISA STAT FAX2100 washer, shaker and microplate reader was used for determination of insulin level.

### 2.4 Experimental animals and housing conditions

Male rats of Wistar strain (120‐150g) were used as experimental animals in the current investigation. They were obtained from the Animal House of National Research Center (NRC), Doki, Giza, Egypt. They were maintained under observation for about 15 days before the onset of the experiment to exclude any intercurrent infection. The chosen animals were housed in polyproplene cages with good aerated stainless steel covers at temperature of 25 ± 5°C, humidity of 55 ± 5% and normal 12‐hr light/dark cycle as well as under good ventilation and received water and standard balanced diet ad libitum. All animal care and experimental procedures were approved by Ethics Committee for Use and Care of experimental animals, Faculty of Science, Beni‐Suef University, Egypt (Ethical Approval Number: BSU/FS/2016/13). All efforts were made to minimize animal suffering.

### 2.5 Induction of DM

Experimental DM was induced in overnight fasted rats using a single intraperitoneal (i.p.) injection of STZ (60 mg/kg b.w.) dissolved in 0.09 M citrate buffer (pH 4.5), 15 min after the i.p. injection of nicotinamide (120 mg/kg b.w.) prepared in 0.9% saline solution (Aboonabi et al., 2014). Thirty male Wistar rats were injected with NA and STZ. Three injected rats, representing ten percent, were died during the 1st week after injection. Seven days after STZ injection, the remained rats (27 rats) were screened for the hyperglycemic state. Overnight fasted (10–12 hr) rats were supplemented with glucose (3 g/kg b.w.) by oral gavage. After 2‐hr of oral glucose loading, blood samples were taken from lateral tail vein, centrifuged and serum glucose level was measured. Eighteen rats have 2‐hr serum glucose ranging from level 200 to 300 mg/dl were included as diabetic rats in the experiment. No animals died during the course of the experiment after the 1st week of NA/STZ injection.

### 2.6 Animals grouping

After adaptation period, the animals were randomly divided into four groups (each of six rats) as follows:

- Group 1 (Normal control). The rats included in this group were given an equivalent volume of 1% carboxymethylcellulose (CMC) as vehicle, by oral gavage daily for 4 weeks.
- Group 2 (Diabetic control). The rats included in this group were given an equivalent volume of 1% CMC (vehicle), by oral gavage daily for 4 weeks.
- Group 3 (diabetic group treated with *M. paradisica* leaf hydroethanolic extract). The rats within this group were diabetic rats that were daily treated with *M. paradisica* leaf hydroethanolic extract at dose level of 100 mg/kg b.w. by oral gavage (Kadali et al., 2016) for 4 weeks. The dose was dissolved in 1% CMC as a vehicle.
- Group 4 (diabetic group treated with *M. paradisica* peel hydroethanolic extract). The rats included in this group were diabetic rats daily treated with *M. paradisica* peel hydroethanolic extract at dose of 100 mg/kg b.w. by oral gavage (Panigrahi et al., 2017; Zulkifli et al., 2020) for 4 weeks. The dose was dissolved in 1% CMC as a vehicle

### 2.7 Sample collection

By the end of the 4th week, rats were deprived of food for 10–12 hr overnight and anaesthetized with inhalation ethyl ether anaesthesia. Blood samples were collected from jugular vein. Blood samples were left at room temperature to coagulate and then centrifuged at 3,000 rpm for 15 min. The clear non‐hemolysed sera were stored at −20°C pending for analysis of some biochemical parameters.

### 2.8 Tissue preparation

After decapitation and dissection, liver of each rat was quickly excised. Liver was divided into two portions. One portion was homogenized at 4°C in isotonic sterile saline (0.9% NaCl) at concentration 10% (1 g/10 ml). The liver homogenate was centrifuged at 3,000 r.p.m. for five minutes for removing cellular debris. The supernatant of liver homogenate of each rat was separated and stored at −20°C for estimation of glucose‐6‐phosphatase activity and oxidative stress and antioxidant defense system biomarkers. The other portion was kept frozen at −20°C and used for estimation of liver glycogen content.

### 2.9 Biochemical examinations

On the day before sacrifice, blood samples were obtained from lateral tail vein of rats deprived of food overnight and at 2 hr following the administration of glucose solution (3 g/kg b.w.). Blood samples were left to coagulate, centrifuged and clear sera were obtained for determination of glucose concentration according to the method of Trinder (1969). Serum fasting insulin level was assayed by Sandwich ELISA using kits purchased from Linco Research (USA), according to the manufacturer's instructions. Glycogen content in the liver was assayed following the method of Seifter et al. (1950). Liver glucose‐6‐phosphatase activity was determined according to the methods of Begum et al. (1978).

Serum triglycerides (Fossati & Prencipe, 1982), cholesterol (Deeg & Ziegenhorn, 1983), HDL‐cholesterol Burstein et al., 1970) were also estimated. Serum LDL‐cholesterol level was calculated from Friedewald (1972) formula as follow:

$$\mathrm{LDL}\mathrm{cholesterol}=\mathrm{Total}\mathrm{cholesterol}‐\frac{\mathrm{Triglycerides}}{5}‐\mathrm{HDL}\mathrm{Cholesterol}$$

Serum vLDL‐cholesterol concentration was calculated according to Nobert (1995) formula as follow:

$$\mathrm{vLDL}\mathrm{cholesterol}=\frac{\mathrm{Triglycerides}}{5}$$

Serum AST (Murray, 1984), LDH (Buhl & Jackson, 1978) and CK‐MB (Gerhardt, 1977) activities were also estimated using commercial diagnostic kits.

Cardiovascular indices were calculated according to Ross (1992) as follow:

$$\mathrm{Cardiovascular}\mathrm{index}1=\frac{\mathrm{Total}\mathrm{cholesterol}}{\mathrm{HDL}\mathrm{cholesterol}}$$

$$\mathrm{cardiovascular}\mathrm{index}2=\frac{\mathrm{LDL}\mathrm{cholesterol}}{\mathrm{HDL}\mathrm{cholesterol}}$$

LPO level in liver homogenate was assayed by measurement of malondialdehyde (MDA) formation according to the method of Preuss et al. (1998). In brief, the protein was precipitated by adding 0.15 ml 76% trichloroacetic acid (TCA) to 1 ml liver homogenate. Then, 0.35 ml of thiobarbituric acid (TBA) was added, as a colour‐developing agent, to the separated supernatant. The developed faint pink colour was measured at 532 nm after incubation in water bath at 80°C for 30 min. MDA (1,1,3,3‐tetramethoxypropane) was used as standard.

GSH content in liver was determined by adding 0.5 ml 5,5'‐Dithiobis(2‐nitrobenzoic acid), Ellman's reagent (as a colour‐developing agent) and phosphate buffer solution (pH 7) to homogenate supernatant after protein precipitation based on the procedure of Beutler et al. (1963). The developed yellow colour in samples and GSH standard was measured at 412 nm against blank.

Liver SOD activity was determined according to the method of Marklund and Marklund (1974). The reaction is based on the inhibition of auto‐oxidation of pyrogallol by SOD. The process is dependent on the presence of superoxide ions. The amount of the enzyme that causes a 50% inhibition in the extinction changes in 1 min compared to the control is regarded as one unit of the enzyme. Briefly, 50 µl of pyrogallol (10 mM) was added to 1 ml of the homogenate supernatant in the presence of Tris buffer (pH 8). The initial absorbance was measured after addition of pyrogallol and at 10 min after addition. The inhibition of the developed yellow colour at 430 nm and the enzyme activity were calculated.

Liver GST activity was assayed according to the method of Habig et al. (1974) in the presence of GSH and 1‐chloro‐2,4‐dinitrobenzene (CDNB) dissolved in ethanol. Calculations were made by using a molar extinction coefficient of 9.6 mM^‐1^ cm^‐1^. In brief, 250 µl CDNB (4 mM) was added to a Wasserman tube containing 250 µl sample, 250 µl GSH solution (4 mM) and 250 µl phosphate buffer (pH 7.3). The developed colour was measured after 10 min of incubation at 25°C.

Liver GPx activity was measured according to the procedure of Matkovics et al. (1998) based on the detection of the GSH that was converted to oxidized glutathione (GSSG) by the enzyme through detection of the residual GSH and subtracting it from the total. Briefly, 50 µl homogenate supernatant was added to a Wasserman tube containing 350 µl Tris buffer (pH 7.6), 50 µl GSH solution (2 mM) and 50 µl H_2_O_2_ (3.38 mM). Then, after 10 min of incubation, the residual GSH content was measured by the previously described method for GSH determination at 430 nm. Standard test was prepared by adding 50 µl distilled water instead of 50 µl sample and blank test was prepared by adding 100 µl distilled water instead of 50 µl sample and 50 µl GSH solution. After detection of residual GSH content in the sample, the GSH converted to oxidized form (GSSG) and the enzyme activity can be calculated.

Because abnormalities in insulin action are poorly detected by a single determination of glucose or insulin levels (Laakso, 1993), the insulin sensitivity was evaluated by homeostasis model assessment estimate of insulin sensitivity (HOMA‐IS) (Aref et al., 2013; Mishra et al., 2018) as follow:

$$\mathrm{HOMA}‐\mathrm{IS}=\frac{10,000}{\mathrm{fasting}\mathrm{insulin}(\mu \mathrm{IU}/\mathrm{ml})\times \mathrm{fasting}\mathrm{glucose}(\mathrm{mg}/\mathrm{dl})/18}$$

HOMA‐β cell function was also calculated (Kuang et al., 2015) using formula as follow:

$$\mathrm{HOMA}‐\beta \mathrm{cell}\mathrm{function}=\frac{20\times \mathrm{fasting}\mathrm{insulin}(\mu \mathrm{IU}/\mathrm{ml})}{[\mathrm{fasting}\mathrm{glucose}(\mathrm{mg}/\mathrm{dl})/18]‐3.5}$$

### 2.10 Statistical analysis

Statistical analysis was performed using Statistical Package for Software Package, SPSS version 20. Results were represented as mean ± standard error (SE) and all statistical comparisons were made by means of one‐way ANOVA test followed by Tukey's test post hoc analysis. The *p* values >.05 were considered non‐significantly different while those of *p* < .05, *p* < .01 and *p* < .001 were significantly, highly significantly and very highly significantly different, respectively..

## 3 RESULTS

NA/STZ‐induced diabetic rats showed a very highly significant elevation (*p* < .001) in fasting and postprandial blood glucose levels after oral glucose loading (3 glucose/kg b.w.) as compared to normal rats (Table 1). Oral administration of either *M. paradisiaca* leaf or peel hydroethanolic extracts to diabetic rats significantly (*p* < .001) decreased the elevated blood glucose levels.

**TABLE 1:** Fasting and postprandial serum glucose level in normal, diabetic control and diabetic rats treated with *M. paradisiaca* leaf and peel hydroethanolic extracts

Variables	Normal	Diabetic control	Diabetic group treated with leaf extract	Diabetic group treated with peel extract
Fasting serum glucose (mg/dl)	90.11 ± 1.55	263.95 ± 6.93^+++^	122.83 ± 2.18^***^	133.50 ± 1.72^***^
Serum glucose after 2hr oral glucose loading (mg/dl)	101.11 ± 2.76	317.83 ± 2.00^+++^	155.50 ± 1.89^***^	143.33 ± 1.45^***^

Note

Data are expressed mean ± SE. Number of animals in each group is six. Values were considered significantly different at ^+++^ *p* < .001 versus control and ^***^ *p* < .001 versus diabetic group.

Diabetic group of rats showed a very highly significant (*p* < .001; LSD) decline in serum insulin, HOMA‐IS and HOMA‐β cell function when compared with normal control. Oral treatment of diabetic rats with *M. paradisiaca* leaf extract as well as peel extract significantly (*p* < .001) increased the diminished serum insulin level, HOMA‐IS and HOMA‐β cell function (Table 2).

**TABLE 2:** Serum insulin level, HOMA‐IS and HOMA‐β cell function in normal, diabetic control and diabetic rats treated with *M. paradisiaca* leaf and peel hydroethanolic extracts

Variables	Normal	Diabetic control	Diabetic group treated with leaf extract	Diabetic group treated with peel extract
Insulin (μU/ml)	24.41 ± 0.86	13.16 ± 0.31^+++^	17.12 ± 0.29^***^	16.58 ± 0.19^***^
HOMA‐IS	82.84 ± 3.84	52.18 ± 2.14^+++^	88.61 ± 2.26^***^	78.95 ± 1.85^***^
HOMA‐β cell function	329.28 ± 16.65	23.69 ± 0.76^+++^	100.53 ± 3.49^***^	87.98 ± 2.53 ^***^

Note

Data are expressed mean ± SE. Number of animals in each group is six. Values were considered significantly different at ^+++^ *p* < .001 versus control and ^***^ *p* < .001 versus diabetic group. HOMA‐IS: Homeostasis Model Assessment‐Insulin Sensitivity; HOMA‐β cell function: Homeostasis Model Assessment‐β cell function.

Concerning liver glycogen content, diabetic rats exhibited a very highly significant (*p* < .001) decrease when compared with normal rats. The treatment of NA/ STZ‐induced diabetic rats with *M. paradisiaca* leaf or peel hydroethanolic extracts produced a very highly significant increase (*p* < .001) in the lowered glycogen content (Table 3).

**TABLE 3:** Liver glycogen content and glucose‐6‐phosphatase activity in normal, diabetic control and diabetic rats treated with *M. paradisiaca* leaf and peel hydroethanolic extracts

Variables	Normal	Diabetic control	Diabetic group treated with leaf extract	Diabetic group treated with peel extract
Liver glycogen content (mg/g tissue)	24.72 ± 1.35	10.12 ± 0.30^+++^	16.76 ± 0.69^***^	14.44 ± 0.51^***^
Glucose−6‐phosphatase activity (mg pi liberated/100 mg tissue/hr)	4.09 ± 0.20	10.96 ± 0.41^+++^	7.11 ± 0.27^***^	7.45 ± 0.16^***^

Note

Data are expressed mean ± SE. Number of animals in each group is six. Values were considered significantly different at ^+++^ *p* < .001 versus control and ^***^ *p* < .001 versus diabetic group.

Liver glucose‐6‐phosphatase activity, on the other hand, was very highly significantly elevated (P˂0.001) in NA/STZ‐induced diabetic rats as compared with normal rats. The treatment of NA/STZ‐induced diabetic rats with *M. paradisiaca* leaf and peel hydroethanolic extracts produced a very highly significant (*p* ˂ .001) decrease in the elevated enzyme activity (Table 3).

Data regarding the effect of *M. paradisiaca* leaf and peel hydroethanolic extracts on lipid profile of diabetic rats are presented in Table 4. Diabetic rats exhibited significant increase (*p* < .001) in serum total cholesterol, triglycerides, LDL‐cholesterol, vLDL‐cholesterol when compared to the normal control group. In contrast, HDL‐cholesterol was affected in an opposite manner, as it was significantly (*p* < .001) lowered in diabetic rats. The administration of *M. paradisiaca* leaf and peel hydroethanolic extracts led to a very highly significant (*p* < .001) amelioration of all parameters of the altered lipid profile. As indicated in Table 4, the treatment with *M. paradisiaca* peel extract was more potent than leaf extract in improving the deteriorated effects of NA/STZ diabetes on serum lipid profile.

**TABLE 4:** Serum lipid profile and cardiovascular risk indices in normal, diabetic control and diabetic rats treated with *M. paradisiaca* leaf and peel hydroethanolic extracts

Variable	Normal	Diabetic control	Diabetic group treated with leaf extract	Diabetic group treated with peel extract
Total cholesterol (mg/dl)	73.16 ± 4.78	112.51 ± 7.81^+++^	78.50 ± 4.47^***^	73.66 ± 3.40^***^
Triglycerides (mg/dl)	77.16 ± 3.57	134.01 ± 4.77^+++^	90.16 ± 2.54^***^	84.16 ± 3.43^***^
HDL‐cholesterol (mg/dl)	43.83 ± 1.35	27.33 ± 0.88^+++^	35.60 ± 1.04^***^	37.43 ± 1.11^***^
LDL‐cholesterol (mg/dl)	20.03 ± 1.29	83.33 ± 4.67^+++^	38.84 ± 2.42^***^	30.31 ± 2.04^***^
vLDL‐cholesterol (mg/dl)	14.63 ± 0.95	22.82 ± 1.59^+++^	15.70 ± 0.89^***^	14.73 ± 0.76^***^
Total‐cholesterol/ HDL‐cholesterol	1.77 ± 0.12	4.91 ± 0.17^+++^	2.22 ± 0.16^***^	1.96 ± 0.11^***^
LDL‐cholesterol/ HDL‐cholesterol	0.44 ± 0.01	3.05 ± 0.18^+++^	1.08 ± 0.06^***^	0.80 ± 0.05^***^

Note

Data are expressed mean ± SE. Number of animals in each group is six. Values were considered significantly different at ^+++^ *p* < .001 versus control and ^***^ *p* < .001 versus diabetic group. HDL‐cholesterol: high density lipoprotein‐cholesterol; LDL‐cholesterol: low density lipoprotein‐cholesterol; vLDL‐cholesterol: very low density lipoprotein‐cholesterol.

The ratios of total cholesterol and LDL‐cholesterol to HDL‐cholesterol were significantly (*p* < .001) increased in diabetic rats as compared to normal control group. *M. paradisiaca* leaf as well as peel hydroethanolic extracts produced a very highly significant (*p* < .001) decrease in these altered values; the treatment with *M. paradisiaca* peel extract was more effective than leaf extract in decreasing the elevated ratios (Table 4).

Serum cardiac function biomarkers, CK‐MB, LDH and AST activities were deleteriously increased (*p* < .001) in diabetic control rats. The treatment of diabetic animals with both *M. paradisiaca* extracts induced a highly significant (*p* < .01) decrease in the elevated CK‐MB activity and a very highly significant (*p* < .001) decrease in serum LDH and AST activities as compared with the diabetic control (Table 5). The treatment with peel extract was more effective than leaf extract in decreasing the elevated serum CK‐MB, LDH and AST activities.

**TABLE 5:** Cardiac enzymes in serum of normal, diabetic control and diabetic rats treated with *M. paradisiaca* leaf and peel hydroethanolic extracts

Variables	Normal	Diabetic control	Diabetic group treated with leaf extract	Diabetic group treated with peel extract
CK‐MB (U/L)	348.11 ± 29.26	647.66 ± 50.57^+++^	473.33 ± 39.48^**^	443.33 ± 36.30^**^
LDH (U/L)	1,700.00 ± 70.08	2072 ± 19.15^+++^	1536.33 ± 9.28^***^	1,467.16 ± 70.79^***^
AST (U/L)	159.80 ± 7.27	227.16 ± 9.90^+++^	178.33 ± 1.69^***^	174.33 ± 4.17^***^

Note

Data are expressed mean ± SE. Number of animals in each group is six. Values were considered significantly different at ^+++^ *p* < .001 versus control and ^**^ *p* < .01 & ^***^ *p* < .001 versus diabetic group. CK‐MB: creatine kinase‐MB; LDH: lactate dehydrogenase; AST: aspartate aminotransferase.

The changes in LPO and antioxidant defense system biomarkers including GSH content and SOD, GPx and GST activities in liver were represented in Table 6. The present data indicated that diabetic rats exhibited a very highly significant (*p* < .001) increase in liver LPO represented by MDA production as compared to the normal control group. The administration of *M. paradisiaca* leaf and peel hydroethanolic extracts induced a very highly significant (*p* < .001) decrease in the elevated LPO when compared to untreated diabetic control (Table 6).

**TABLE 6:** Oxidative stress and antioxidant defense markers in liver normal, diabetic control and diabetic rats treated with *M. paradisiaca* leaf and peel hydroethanolic extracts

Variables	Normal	Diabetic control	Diabetic group treated with leaf extract	Diabetic group treated with peel extract
LPO (nmole MDA/100 mg tissue/hr)	29.09 ± 1.10	65.39 ± 2.89^+++^	49.60 ± 2.75^***^	38.70 ± 3.29^***^
GSH (nmole/100 mg tissue)	25.07 ± 1.00	11.22 ± 0.30^+++^	15.73 ± 0.94^***^	18.80 ± 0.67^***^
SOD (U/g tissue)	14.09 ± 0.62	5.14 ± 0.48^+++^	9.03 ± 0.39^***^	10.01 ± 0.39^***^
GPx (mU/100 mg tissue)	186.76 ± 1.11	158.60 ± 2.37^+++^	175.82 ± 1.48^***^	179.23 ± 2.02^***^
GST (U/100 mg tissue)	181.77 ± 5.67	151.74 ± 1.56^+++^	169.22 ± 1.28^***^	173.91 ± 0.23^***^

Note

Data are expressed mean ± SE. Number of animals in each group is six. Values were considered significantly different at ^+++^ *p* < .001 versus control and ^***^ *p* < .001 versus diabetic group. LPO: lipid peroxidation; GSH: reduced glutathione; SOD: superoxide dismutase; GPx: glutathione peroxidase; GST: glutathione‐S‐transferase.

On the other hand, the diabetic control exhibited a very highly significant (*p* < .001) decrease in liver GSH content and SOD, GPx and GST activities in comparison with the normal control. Meanwhile, *M. paradisiaca* leaf as well as peel extracts treated diabetic groups exhibited a very highly significant (*p* < .001) increase in the lowered values of these antioxidants when compared to untreated diabetic control group (Table 6).

The treatment with peel extract was more effective than leaf extract in improving the deteriorated changes in LPO, GSH content and antioxidant enzyme activities in the diabetic rats (Table 6).

## 4 DISCUSSION

The herbal anti‐diabetic drugs are yet to be commercially formulated as modern medicines, even though they have been acclaimed for their therapeutic properties in the traditional systems of medicine (Wadkar et al., 2008). In NA/STZ‐induced diabetic animals, the present data indicate a marked increase in serum fasting and postprandial glucose levels associated with suppressed insulin sensitivity (HOMA‐IR) and impaired β‐cell function (HOMA‐β cell function) when compared to normal rats. These results are in line with Ahmed, et al. (2017).

Based on the GC‐MS analysis of the hydroethanolic of *M. paradisiaca peel* and leaf, many phytochemicals have been present which contributes to the medicinal activity of the plant. The major components which present in the leaves of the plant *M. paradisiaca* and other *Musa species* were n‐hexadecanoic acid, phytol and 9,12,15‐octadecatrienoic acid, methyl ester, (Z,Z,Z)‐ while fruit peels contain high percentage of vitamin E, estragole, hexadecanoic acid ethyl, epicatechin, gallocatechin, p‐coumaric acid ethyl ester and oleamide which contribute the activities like anti‐inflammatory, antioxidant, anticancer, hypercholesterolemic, anti‐diabetic, antiulcerogenic and others (Abdel Aziz et al., 2020; Duke, 2007; Waghmare et al., 2014).

The present data demonstrated that the treatment of diabetic rats with *M. paradisiaca* leaf and peel hydroethanolic extracts produced a marked decrease in serum glucose at dose of 100 mg/kg b.w. The decrease in elevated serum glucose levels are line with Ajiboye et al. (2018). These hypoglycemic effects might be attributed to contained compounds. GC‐MS analysis of *M. paradisiaca* extract revealed the presence of sterols, phytol, vitamine E, Linoleic acid and plamitc acid. These compounds have been elucidated to exert anti‐hyperglycemic effect by several authors (Alamdari et al., 2018; Kumar et al., 2010; Matsuda et al., 2018; Ragasa et al., 2011).

NA/STZ‐induced diabetic rats in the current study exhibited a significant decrease in circulating fasting insulin levels as well as calculated HOMA‐β cell function, indicating the presence insulin deficiency state. These results are in accordance with other publications (Aboonabi et al., 2014; Ahmed, et al., 2017; Szkudelski, 2012 and b). This might be due to the destruction of the pancreatic β‐cells and thereby induces hyperglycemia (Ahmed, et al., 2017 and b). The calculated HOMA‐IS was also significantly decreased in NA/STZ‐induced diabetic rats, reflecting the presence of impaired tissue insulin sensitivity or insulin resistance. The oral administration of *M. paradisiaca* leaf and peel extracts induced a significant increase in serum insulin level concomitant with increased values of HOMA‐IS and HOMA‐β cell function; thereby the improvement in the glucose homeostasis is plausibly due to an improvement in insulin action and insulin secretion. These results are in agreement with Ojewole and Adewunmi (2003) who reported that the green fruit of *M. paradisiaca* have hypoglycemic effect partly due to stimulation of insulin production and glucose utilization.

Liver plays a central role in buffering the postprandial hyperglycemia and is involved in synthesis of glycogen which is the primary intracellular form in which glucose is stored. Glycogen levels in different tissues, especially the liver, are a direct indication of insulin activity as insulin hormone stimulates intracellular glycogen synthesis by activating glycogen synthetase and inhibiting glycogen phosphorylase (Grover et al., 2000). Our results demonstrated a significant depletion in hepatic glycogen content associated with significant increase in glucose‐6‐phosphatase activity. These changes are in concurrence with those of Vats et al. (2003) and Ahmed, et al. (2017) who found that STZ‐induced diabetes reduced hepatic glycogen content and increased glucose‐6‐phosphatase activity in diabetic rats. In addition, Musabayane et al. (2005) reported that DM is associated with a marked decrease in the levels of liver glycogen. Also, these results are in agreement with the work of Grover et al. (2000). The reduced glycogen store has been attributed to the reduction in the activity of glycogen synthase and an increase in the activity of glycogen phophorylase as result of insulin deficiency (Larner, 1975) which in turn results in the activation of glycogenolytic and gluconeogenic pathways (Abdel‐Moneim et al., 2001; Vats et al., 2004). NA/STZ‐induced diabetic rats that were treated with *M. paradisiaca* leaf and peel hydroethanolic extracts positively modulated the decrease of hepatic glycogen content. These results are in line with Ajiboye et al. (2018) who stated that diabetic rats placed on *M. paradisiaca*‐based diet exhibited increase in liver glycogen content and serum insulin and decrease in the activity of glucose6‐phosphatase.

Lipids play a crucial role in the pathogenesis of DM and the most common lipid abnormalities in DM are hypercholesterolemia and hypertriglyceridemia (Palumbo, 1998) and secondary elevation of free fatty acids' level in the blood (Singh et al., 1987). In the present study, the increase in serum glucose level was accompanied by a marked elevation in total cholesterol, LDL‐cholesterol, triglycerides and reduction in HDL‐cholesterol in NA/STZ‐induced diabetic rats. These obtained results are in concordance with those of Ahmed, et al. (2017) and Pierre et al. (2012). In addition, these results are consistent with Arkkila et al. (2001) who revealed that the abnormalities in the lipid profile may be due to insulin deficiency. Furthermore, similar results were obtained in several studies in animal or experimental DM (Chertow, 2004; Cullen et al., 1999). Sout (2005) considered diabetic deregulation of blood lipid levels and hyperglycaemia to be considerable risks for cardiovascular complications. The treatments of diabetic rats with *M. paradisiaca* leaf and peel hydroethanolic extracts, in the present study, reversed the abnormalities in serum lipid profile and the increased ratios of total cholesterol and LDL‐cholesterol to HDL‐cholesterol, probably by enhancing the insulin secretion and improving the insulin sensitivity as well. The significant increase in the calculated HOMA‐β cell function and HOMA‐IS in the diabetic rats treated with *M. paradisiaca* leaf and peel hydroethanolic extracts as compared with diabetic control supports this attribution. The hypolipdemic effect may also be due to the presence of polyunsaturated fatty acids, linoleic acid ester and vitamin E in the tested extracts (Kumar et al., 2010).

In addition, total cholesterol/HDL‐cholesterol and LDL‐cholesterol/HDL‐cholesterol have been used to predict risks of cardiovascular abnormalities (Grover et al., 1999). These findings were confirmed by the elevated serum activities of CK‐MB, LDH and AST. Supplementation with either *M. paradisiaca* leaf or peel hydroethanolic extract might lead to reduction in the risk of developing vascular and heart diseases. The observed cardio‐protective effects of both tested *M. paradisiaca* extracts was further confirmed by the notably decreased serum cardiac markers, CK‐MB, LDH and AST.

In view of oxidative stress, liver LPO increased while GSH content and antioxidant enzyme (SOD, GPx and GST) activities decreased in diabetic rats of the present study. These data are consistent with Damasceno et al. (2002), Gul et al. (2002) and Ahmed et al. (2020) who reported that STZ produces oxidative stress and depletion of antioxidant systems in both blood and tissues. Also, these results were in accordance with other reports revealing an increase in lipid hydroperoxides in the plasma of diabetic subjects (Ahmed, 2005; Kakkar et al., 1995) and in animals with experimental DM (Desco et al., 2002; Ramesh & Pugalendi, 2005). It is worth mentioning that both diabetic subjects and experimental animal models exhibit an increase in the oxidative stress owing to persistent and chronic hyperglycemia together with depletion in the activity of the anti‐oxidative defense system, thereby promoting free radical production (Ahmed et al., 2020; Baynes & Thorpe, 1996). From the other hand, the decrease in antioxidant enzyme activity under diabetic conditions could be attributed to the glycation of these enzymes, which occurred at persistently elevated blood glucose levels (Taniguchi, 1992). Rats treated with *M. paradisiaca* leaf and peel hydroethanolic extracts were able to restore the level of GSH and activities of GPx, SOD and GST and successfully produced a significant suppression of LPO in diabetic rats. These results are in line with Mokbel and Hashinaga (2005) and who reported that antioxidant activity of aqueous acetone extract of banana peel. Vijayakumar et al. (2008) reported the antioxidant activity of the extracted flavonoids from *M. paradisiaca* in rats. According to GC‐MS results of the extract, the presence of vitamine E and palmitic acid, which are already known to possess high antioxidant activities, may be related to the greater antioxidant activity (Almeida et al., 2012; Elagbar et al., 2016; Kaplaner et al., 2017; Varvařovská et al., 2004).

## 5 CONCLUSION

The present study revealed that *M. paradisiaca* leaf and peel hydroethanolic extracts possess anti‐hyperlipidemic and cardioprotective effect in NA/STZ‐induced diabetic rats; the effect of *M. paradisiaca* peel hydroethanolic extract was more potent. These improvements may be attributed to alleviation in the glycemic state, β‐cell insulin secretory response, tissue insulin sensitivity and antioxidant defense system.

## 6 COMMISION OF ETHICS AND ANIMAL WEFARE

The authors confirm that the ethical policies of the journal, as noted on the journal's author guidelines page, have been adhered to and the appropriate ethical review committee approval has been received. All animal care and experimental procedures were approved by Ethics Committee for Use and Care of Experimental Animals, Faculty of Science, Beni‐Suef University, Egypt (Ethical Approval Number: BSU/FS/2016/13). All efforts were made to minimize animal suffering.

## CONFLICT OF INTEREST

The authors declare that they have no conflict of interest.

## AUTHOR CONTRIBUTIONS

**Osama M. Ahmed:** Conceptualization; Data curation; Formal analysis; Methodology; Project administration; Supervision; Validation; Visualization; Writing‐review & editing. **Sanaa Abd El‐Twab:** Conceptualization; Data curation; Investigation; Methodology; Supervision; Visualization; Writing‐original draft. **Hessah M. Al‐Muzafar:** Funding acquisition; Project administration; Resources; Visualization; Writing‐review & editing. **Kamal A Amin:** Funding acquisition; Project administration; Validation; Visualization; Writing‐review & editing. **Sarah Abdel Aziz:** Data curation; Formal analysis; Investigation; Methodology; Resources; Software; Visualization; Writing‐original draft. **Mohammed Abdel‐Gabbar:** Conceptualization; Data curation; Formal analysis; Investigation; Project administration; Supervision; Validation; Writing‐review & editing.

### PEER REVIEW

The peer review history for this article is available at https://publons.com/publon/10.1002/vms3.389.

## Data Availability

All data described in the manuscript are found in tables 1‐7 that were added in the submitted article below references.
